# Supplementary material for: Crystal structure of E. coli apolipoprotein N-acyl transferase
Source: Nat Commun. 2017 Jul 4;8:15948. doi: 10.1038/ncomms15948 (PMC5500890; doi:10.1038/ncomms15948)
Supplement: Supplementary Information [file ncomms15948-s1.pdf]

File name: Supplementary Information
Description: Supplementary figures, supplementary table and supplementary references.

File name: Supplementary Movie 1
Description: While in sim 1 simulation, a lipid molecule (show as green sticks) enters the cavity during the 150 ns simulation.

File name: Peer review file
Description:

a.

b.

c.

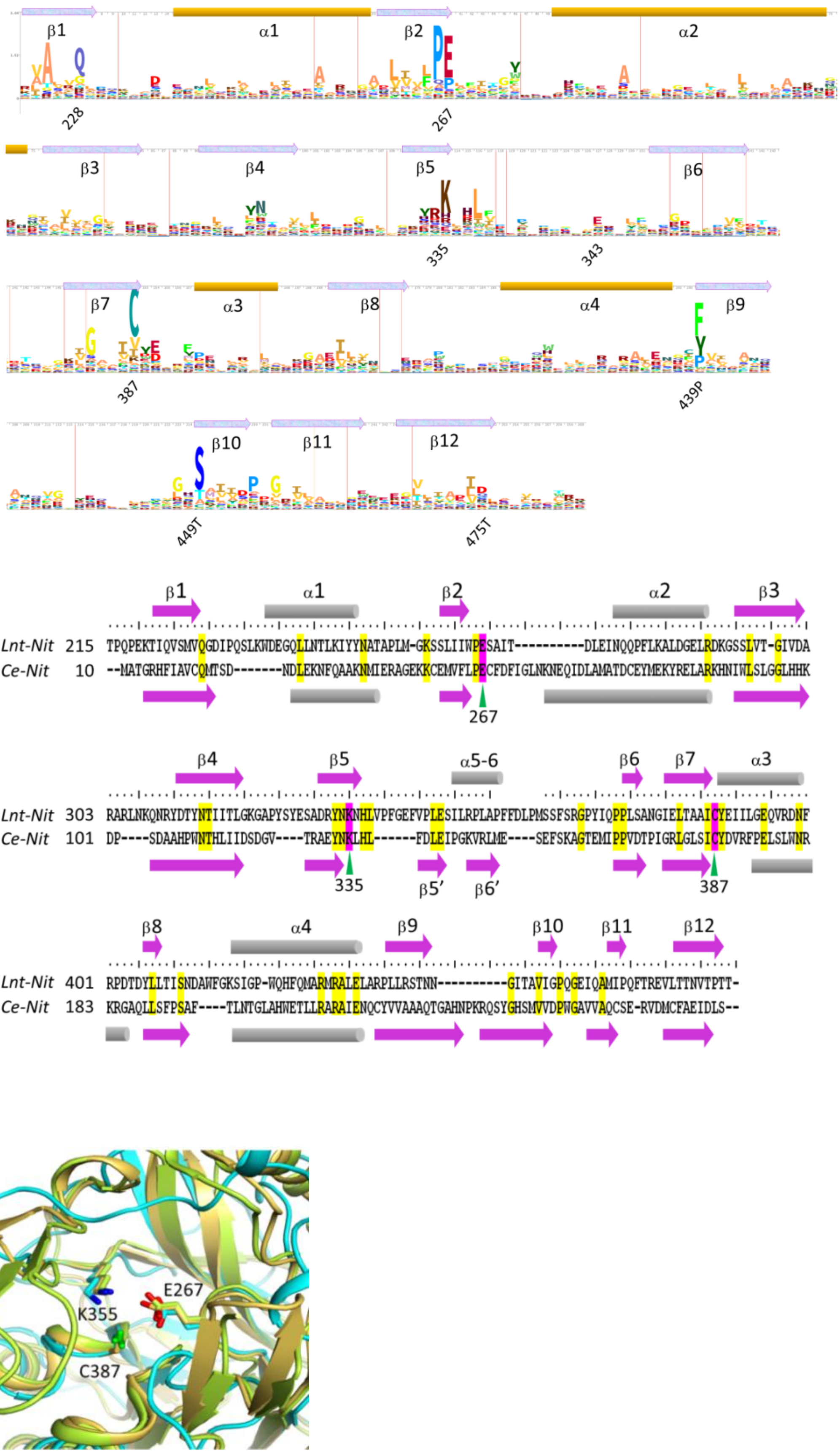
β1
α1
β2
α2
228
267
β3
β4
β5
β6
335
343
β7
α3
β8
α4
β9
387
439P
β10
β11
β12
449T
475T
Lnt-Nit 215
Ce-Nit 10
Lnt-Nit 303
Ce-Nit 101
α5-6
β5′
β6′
Lnt-Nit 401
Ce-Nit 183
E267
K355
C387

Supplementary figure 1. Comparison of the Nit domains of Ec-Lnt and other nitrilases

a. The sequence homology of Nit domains is shown in Logo presentation [1]. Secondary structures are marked on the top. Positions of selected residues from *E. coli* Lnt are marked below the sequence logo. Among the selected residues, E267, K335, and C387 form the catalytic trial; Q228 and S449 form an H-bond; and P439 is responsible for a sharp turn.

b. 3D structure-based sequence alignment between the Nit domains of Ec-Lnt and *C. elegans* Nit-Fhit (Ce-Nit, PDB ID: 1EMS). The catalytic triad residues are highlighted in magenta, and other identical residues are in yellow. Secondary structure elements from both crystal structures are marked on the corresponding sides.

c. Superposition of the catalytic triad with those from other nitrilases. Backbone trace of Ec-Lnt is colored in cyan; Nit-Fhit (PDB ID: 1EMS) in gold color; and yNit2 (4HG3) in lemon color.

a.

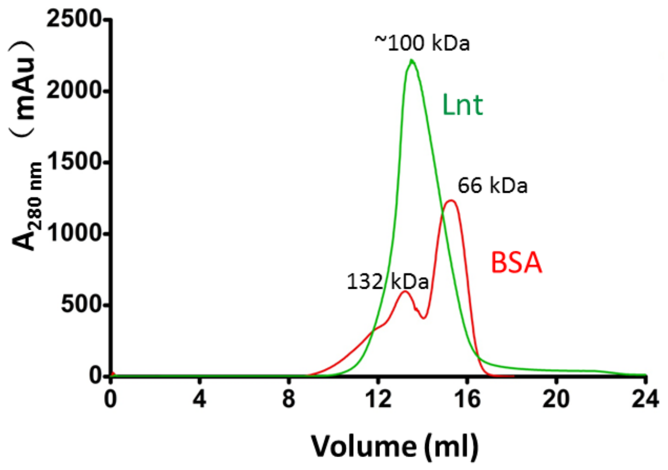

b.

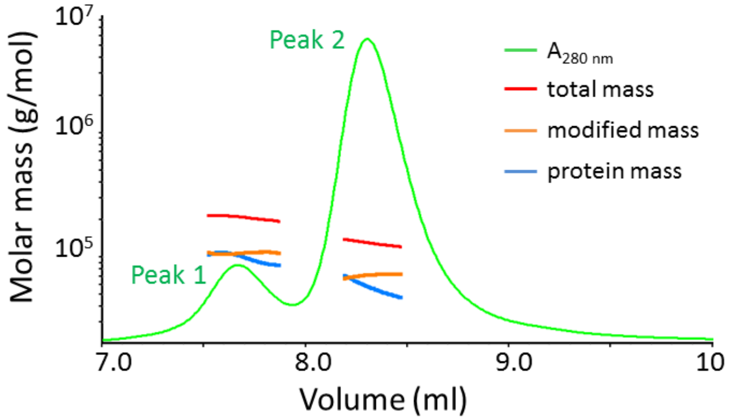

	Peak 1	Peak 2
Total mass: (protein-micelle complex)	207 kDa (±0.1%)	128 kDa (±0.1%)
Protein mass: (measured)	108 kDa (±0.3%)	72 kDa (±0.2%)
(modified)	102 kDa (±0.2%)	73 kDa (±0.2%)
Theoretical mass:	115 kDa (dimer)	58 kDa (monomer)

Supplementary figure 2. Lnt exists as a monomer in solution

a. Result of size-exclusion chromatograph of recombinant Lnt on a Superdex 200 column is shown. Sample of Ec-Lnt protein (10 mg in 1 ml) was injected into the column equilibrated with 25 mM MES (pH 5.5), 100 mM NaCl, and 0.3% (w/v)

NTM. It indicates that Lnt (plus its surrounding detergents) exists as a monomer under the experimental condition used (the green line). Bovine serum albumin (BSA) was used as a molecular marker (the red line).

b. To further measure the oligomeric state of Lnt in solution, the size-exclusion chromatography coupled with multi-angle light scattering (SEC-MALS) method was applied. The system consists of a 1200 series HPLC system (Agilent Technologies), a DAWN HELEOS-II instrument (Wyatt) and an Optilab rEX refractive index instrument (Wyatt). The SEC-MALS system was pre-calibrated with bovine serum albumin protein standard. 20 μL of wild-type Lnt protein sample purified in DM (protein concentration at ~4 mg/ml) was injected into the WTC-030S5 SEC column (Wyatt) and eluted at 0.5 mg/min in a buffer containing 20 mM HEPES (pH 7.5), 150 mM NaCl, and 0.2% DM. The extinction coefficient of protein at 280 nm was calculated from its amino-acid sequence (A_{280}= 1.986 ml/mg/cm). Data collection and analysis were conducted using the Astra 5 software (Wyatt). The specific refractive index (dn/dc) value of protein at 0.185 ml/g and that of DM at 0.147 ml/g were used for data processing [2].

a.

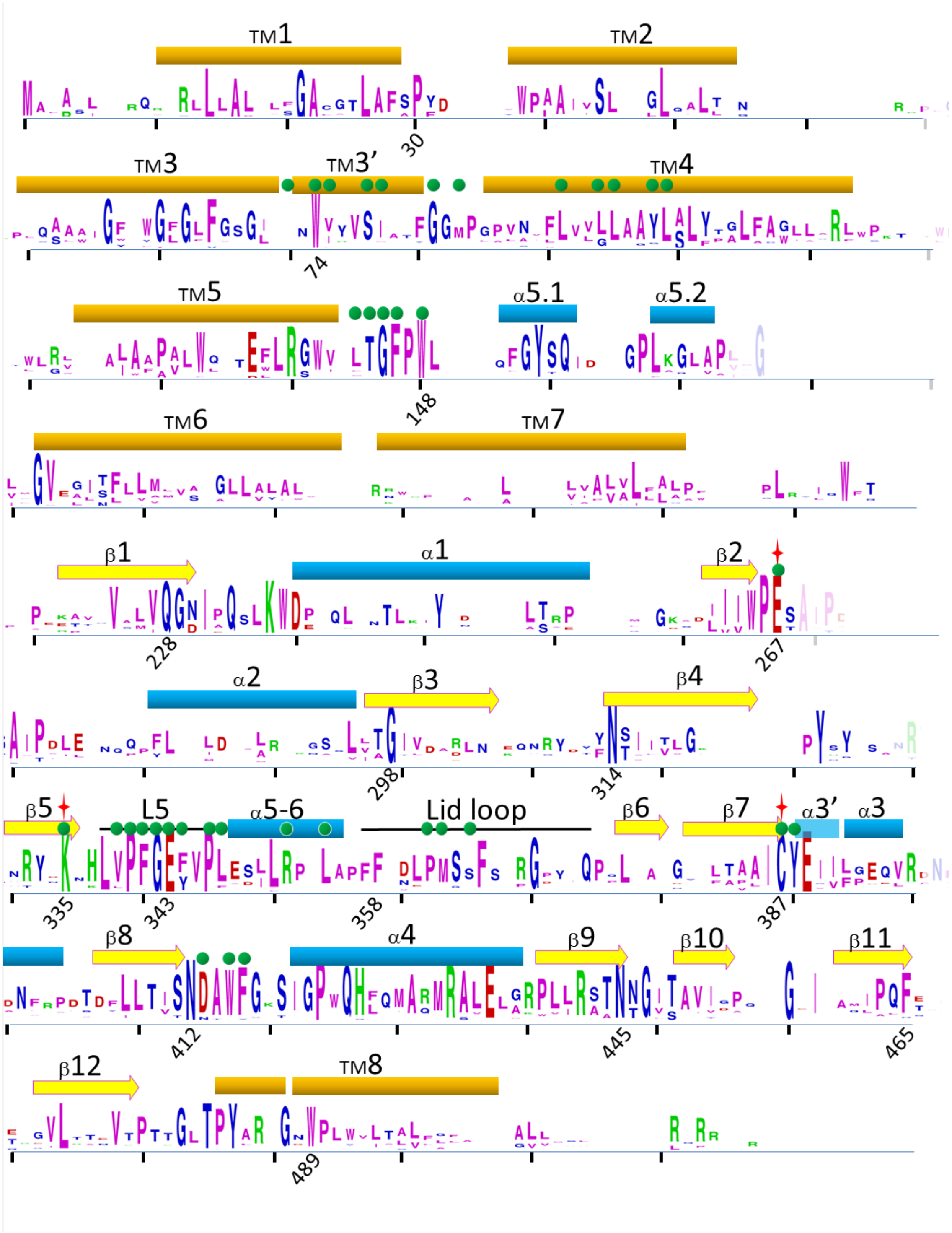

TM1
TM2
30
TM3
TM3’
TM4
74
TM5
α5.1
α5.2
148
TM6
TM7
β1
α1
β2
228
267
α2
β3
β4
298
314
β5
L5
α5-6
Lid loop
β6
β7
α3’
α3
335
343
358
387
β8
α4
β9
β10
β11
412
445
465
β12
TM8
489

b.

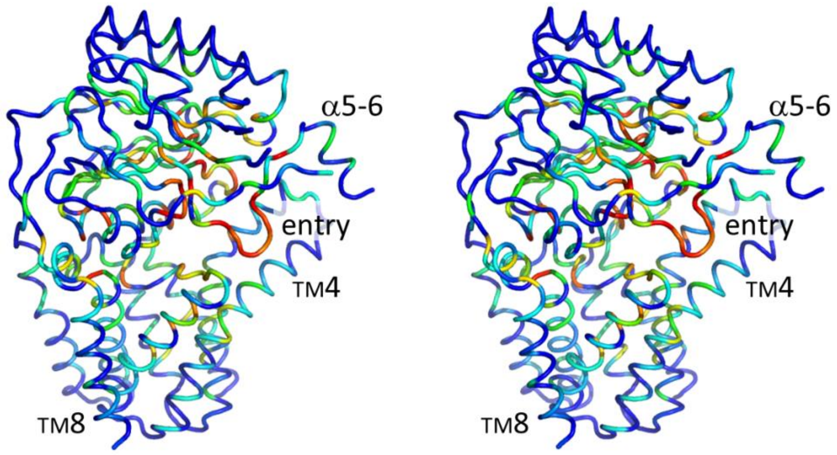

Supplementary figure 3. Homology of the Lnt family

a. The figure of homology logo was generated by the PFAM website, based on alignment of amino acid sequences of 22 Lnt proteins. Hydrophobic, polar, positively charged, negatively charged residues are shown in magenta, blue, green, and red letters, respectively. Ec-Lnt secondary structure elements are marked on the top, with TM helices (TM1–8) represented as orange cylinders, other helices in cyan, and β-strands (β1–12) as yellow arrows. Selected residue numbers of Ec-Lnt are labelled at the bottom. Sites of point mutations are marked with green dots, and catalytic residues are marked with red stars. (The Uniprot IDs of the 22 Lnt proteins used in the alignment are P23930 (*E. coli* Lnt), Q7D7J5, Q8FXT9, Q820C8, Q8D124, Q9ZI86, O24982, O87576, A6V0C5, P74055, B5EGF7, A0KN85, D4GM33, M9RG67, V5KQY2, B2VBL8, D2TNH3, Q0T6R6, C9R373, F0LVH6, E3DK92, and A4SJX0.)

b. Mapping of the conserved residues of the Lnt family into the 3D structure of Ec-Lnt. The Ec-Lnt peptide backbone is shown in a tube presentation in stereo view. Positions from highly conserved to non-conserved are colored from red to blue. Selected secondary structures and the entry of the interfacial opening are labeled.

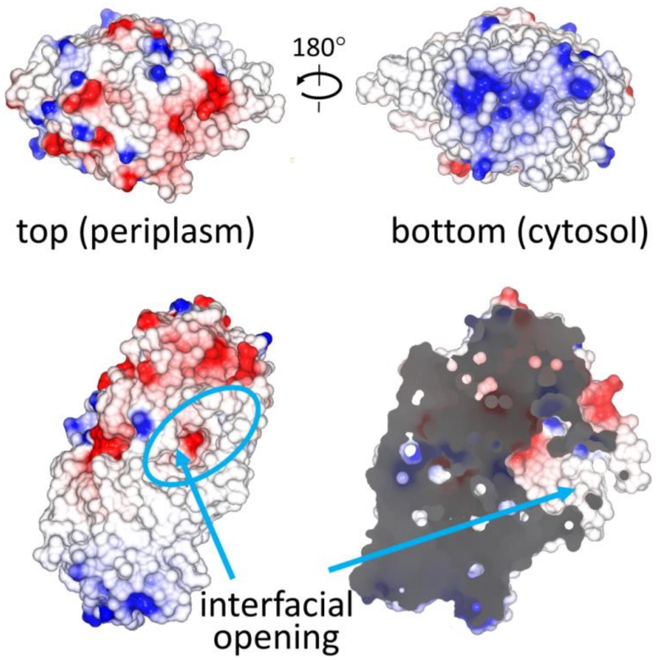

Supplementary figure 4. Surface presentations of electrostatic potential. Positively charged regions are colored in blue, negatively charged regions in red.

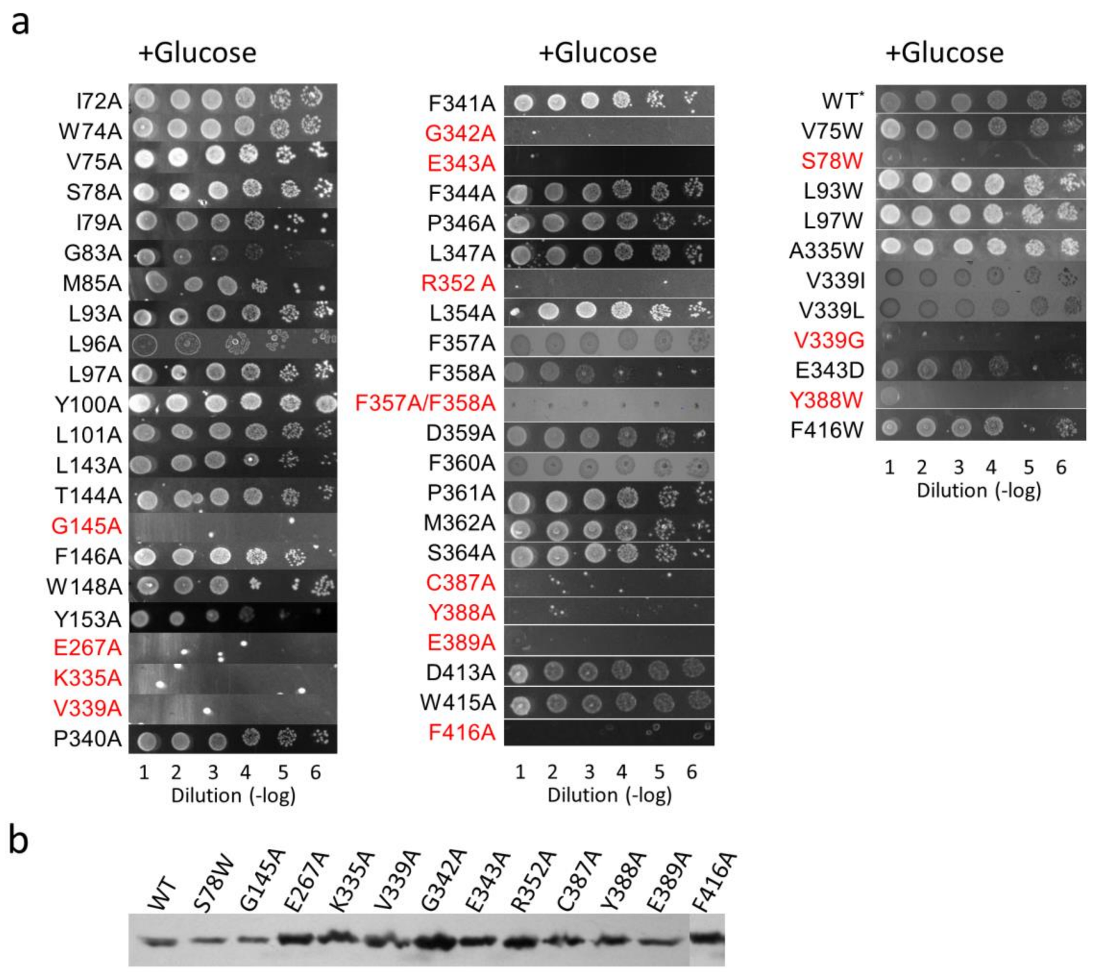

Supplementary figure 5. *In vivo* screening of functionally important residues

a. Results of complementation assays for Lnt variants. For each Lnt variant, a serial dilution of cell culture was spotted onto solid medium containing 11 mM glucose. Photos were taken 16 h after incubation at 37°C. Variants of Lnt that failed to rescue Δ*lnt* are labeled in red ink. (Also see the legend for Figure 1b.)

b. Expression levels of inactive variants compared to WT, verified by using anti-His immunoblotting.

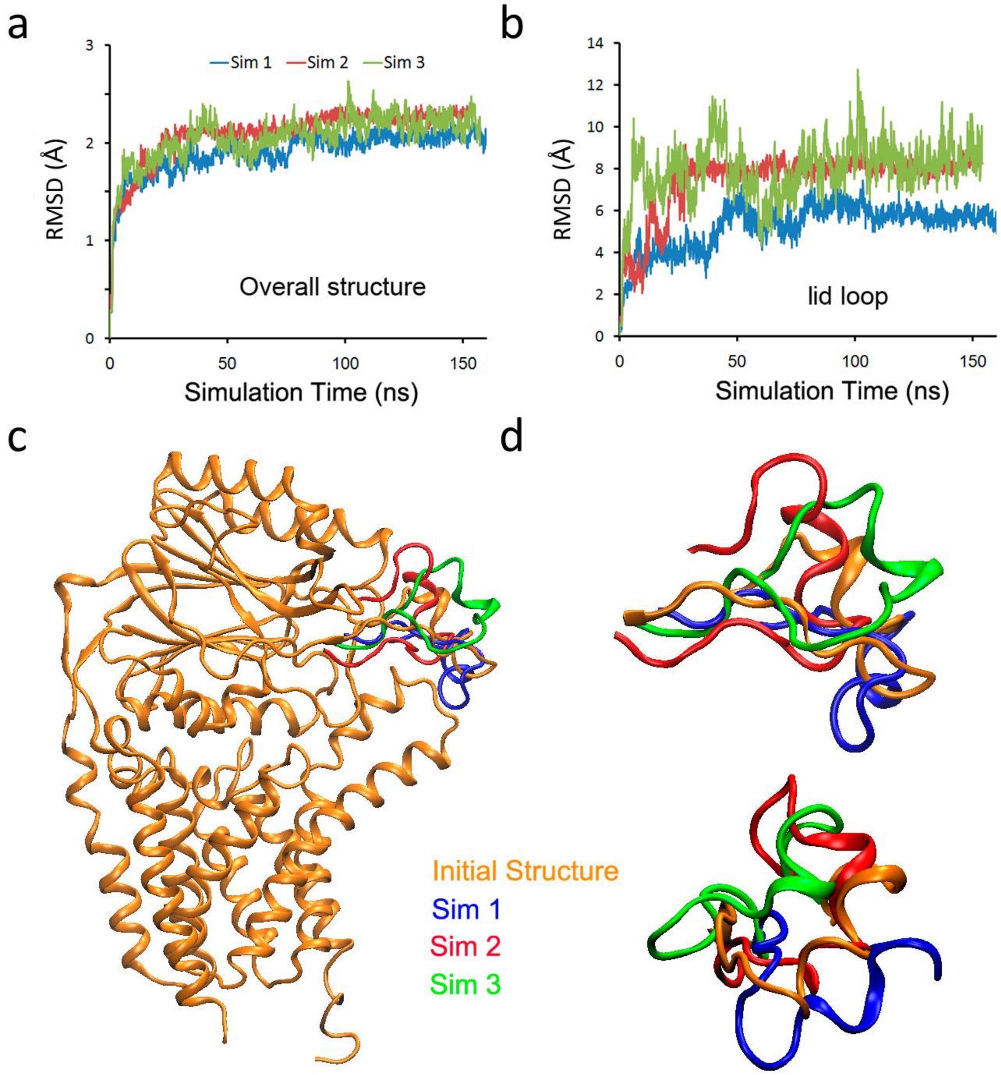

Supplementary figure 6. MD simulations

(a) and (b) Time course of RMSD changes relative to the initial structure for the whole Lnt structure (a) and for the lid loop (b). The lid loop is more flexible than the main body of the enzyme.

(c) and (d) Structure comparison between the initial structure and the last snapshot of the overall structure (c) and zoom-in to the lid loop region (d). In panel c, the whole structure of the initial model is shown, whereas only the lid loop is shown for the last snapshots from the three simulations. In panel d, the two views differ by a 90° rotation around the vertical axis.

a.

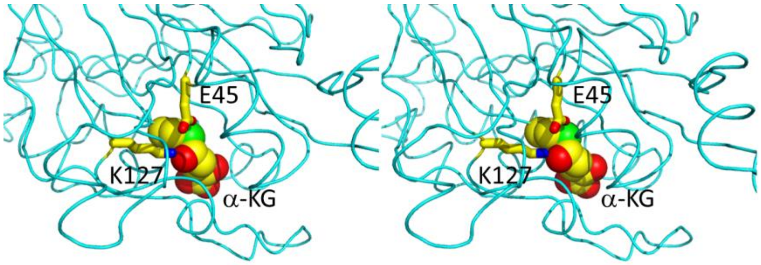

b.

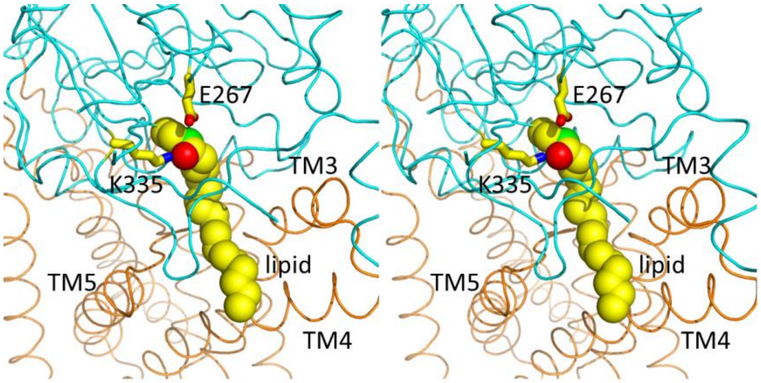

Supplementary figure 7. Putative Lnt-lipid intermediate.

a. Stereo view of the yNit2-αKG intermediate (4HGD) [3].

b. Stereo view of a model of the Lnt-lipid intermediate. The catalytic Cys residue and its ligand are shown in sphere models, and the catalytic Glu and Lys residues are shown in stick models. The Nit domain is shown in cyan, and the TM domain (of Ec-Lnt) in orange.

a.

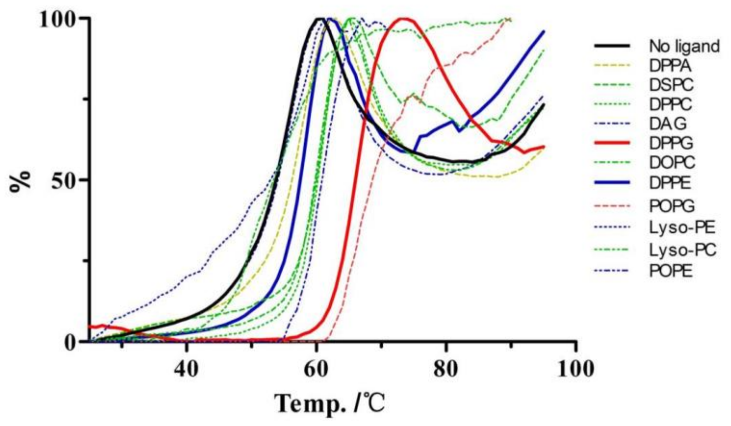

b.

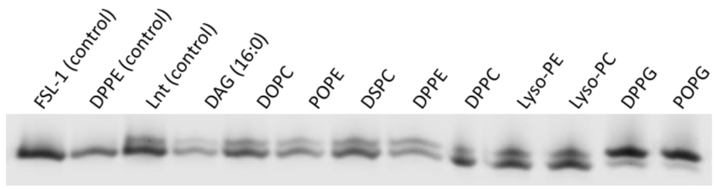

Supplementary figure 8. Interactions of Lnt with different lipids

a. Thermostability Lnt in the presence of different lipids. Thermofluor results of (10 µM) Lnt in the presence of 100 µM of different (additional) lipids are shown. Estimated melting temperatures (Tm), based on fitting to a Boltzmann model, are summarized in Table S1.

b. PG appears to be a good lipid donor for Lnt, as shown in an *in vitro* transacylation assay. Lnt (3.5 nM) was added to a reaction mixture composed of different lipids (2.5 µM) and FSL-1 (40 µM). Samples were incubated for 40 min at 37°C and analyzed by Tricine-SDS-PAGE. The lower and upper bands are unmodified and modified substrates of FSL-1 fluorescein, respectively. The three controls are FSL-1 alone, FSL-1 plus DPPE (labeled as DPPE), and FSL-1 plus Lnt (labeled as Lnt).

DPPA, 1,2-dipalmitoyl-sn-glycero-3-phosphate;

DPPC, 1,2-dipalmitoyl-sn-glycero-3-phosphocholine;

DPPE, 1,2-dihexadecanoyl-sn-glycero-3-phosphoethanolamine;

DPPG, 1,2-dihexadecanoyl-sn-glycero-3-phospho-(1'-rac-glycerol);

POPE, 1-palmitoyl-2-oleoyl-sn-glycero-3-phosphoethanolamine;

POPG, 1-hexadecanoyl-2-(9Z-octadecenoyl)-sn-glycero-3-phospho-(1'-rac-glycerol) (16:0/18:1(9Z));

DOPC, 1,2-dihexadecanoyl-sn-glycero-3-phosphocholine;

DSPC, 1,2-distearoyl-sn-glycero-3-phosphocholine;

Lyso-PE, Lysophosphatidylethanolamine(0:0/16:0);

Lyso-PC, Lysophosphatidylcholine(16:1);

DAG(16:0), diacyl-glycerol (16:0).

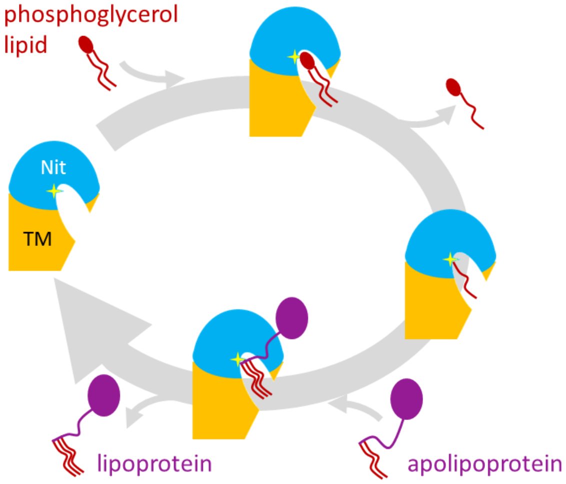

Supplementary figure 9. Schematic diagram of the ping-pong mechanism of the Lnt functional cycle.

The Nit and TM domains are shown in blue and orange, respectively. The catalytic site is marked with a yellow star.

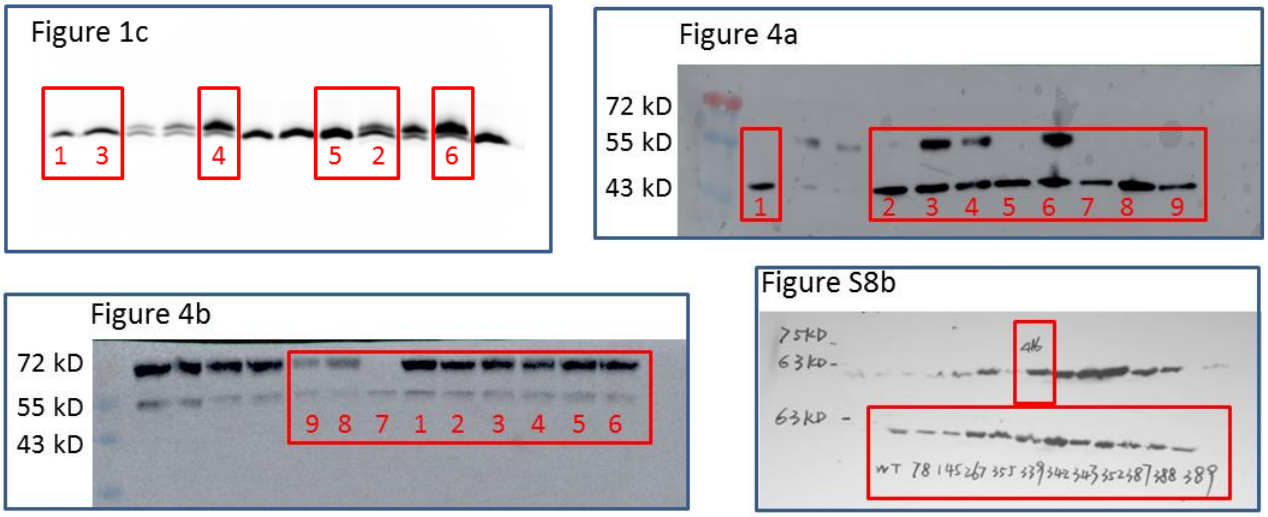

Supplementary figure 10. Uncropped figures of SDS-PAGE gels and Western Blots.

The numbers in red boxes represent corresponding left to right lanes in cropped figures.

Supplementary table 1. Estimated melting temperatures (Tm), based on fitting to the Boltzmann model

Lipid	No ligand	DPPA	DPPC	DPPE	DPPG	POPE	POPG	DOPC	DSPC	Lyso-PE	Lyso-PC	DAG
Tm/°C	51	54	59	56	65	61	69	58	59	58	53	51

Supplementary References

1 Schuster-Bockler, B., Schultz, J. & Rahmann, S. HMM Logos for visualization of protein families. *BMC Bioinformatics* **5**, 7, doi:10.1186/1471-2105-5-7 (2004).

2 Liu, X., Yin, Y., Wu, J. & Liu, Z. Structure and mechanism of an intramembrane liponucleotide synthetase central for phospholipid biosynthesis. *Nat Commun* **5**, 4244, doi:10.1038/ncomms5244 (2014).

3 Liu, H. *et al.* Structures of enzyme-intermediate complexes of yeast Nit2: insights into its catalytic mechanism and different substrate specificity compared with mammalian Nit2. *Acta Crystallogr D Biol Crystallogr* **69**, 1470-1481, doi:10.1107/S0907444913009347 (2013).
